# A Comprehensive Investigation of Potential Bacterial Pathogens in Largemouth Bass (*Micropterus salmoides*)

**DOI:** 10.3390/microorganisms13061413

**Published:** 2025-06-17

**Authors:** Yun-Yao Tu, Qun Lu, Na Zhang, Jie Leng, Qin Yang, Jie Yu, Cheng-Ke Zhu, Tao He, Jian-Yong Hu, Ming-Ji Lv, Song Zhu

**Affiliations:** 1 College of Fisheries, Southwest University, Chongqing 400715, China; 2 Key Laboratory of Freshwater Fish Reproduction and Development (Ministry of Education), Key Laboratory of Aquatic Science of Chongqing, Southwest University, Chongqing 400715, China; 3 Xinjiang Fisheries Research Institute, Scientific Observing and Experimental Station of Fishery Resources and Environment in Northwest China, Ministry of Agriculture, Urumqi 830000, China

**Keywords:** largemouth bass, bacterial pathogens, antibiotic sensitivity, healthful aquaculture

## Abstract

In the study, a comprehensive investigation on potential bacterial pathogens affecting largemouth bass (*Micropterus salmoides*) was performed. Monthly surveys were conducted from April to October 2024. Diseased largemouth bass exhibited diverse clinical symptoms, such as rot of gill and fin, ulcers on body surface, and petechial hemorrhages in liver. Following isolation and identification, a total of 21 potential bacterial pathogens (numbered strain 1 to 21, respectively) were identified. The genus *Aeromonas* had the highest proportion (67.14%), among which the frequency of *Aeromonas veronii* was 24.60%. TEM analysis revealed that the bacterial strains exhibited three predominant shapes (rod-shaped, spherical, and curved) with length ranging from 0.5 to 3 μm. Flagellar structures were observed in strains 1–4, 6–8, 11–17, and 19–21, with variations in number and growth sites. Three isolates (strains 9, 10, 18) demonstrated Gram-positive characteristic, and strains 5, 11, and 18 have capsule structures. Strains 5, 9, 10, and 18 were non-motile, and strains 1–4, 6, 7, 9–11, 16–18, and 21 exhibited β-hemolysis. Physiological and biochemical characteristics of the 21 bacterial isolates were comprehensively analyzed. Antibiotic sensitivity testing revealed that florfenicol and enrofloxacin exhibited excellent antibacterial effects. These data will enrich the potential bacterial diseases information and promote the healthy development of the largemouth bass industry.

## 1. Introduction

Largemouth bass (*Micropterus salmoides*), a freshwater carnivorous fish native to North America’s Mississippi River basin, thrives in low-turbidity lentic or slow-moving aquatic systems [1]. Characterized by rapid growth, strong ecological adaptability, and premium meat quality, this species has emerged as a cornerstone of Chinese aquaculture, with national production exceeding 880,000 metric tons in 2023 [2]. However, aquaculture of largemouth bass is facing escalating disease outbreaks, primarily attributed to genetical characterization decline, blind pursuit of production growth, and widespread antibiotic misuse. These disease challenges have become an important bottleneck restricting the healthy and sustainable development of the largemouth bass farming industry [2,3,4].

Diseases affecting largemouth bass are mainly caused by parasitical, bacterial, and viral pathogens, with bacterial infections constituting the most frequent and economically devastating threat. According to the survey data on the diseases of largemouth bass from 2019 to 2023, bacterial diseases account for 61% of all diseases. A variety of bacteria can infect largemouth bass and cause epidemic diseases, such as *Nocardia seriolae* [2,5,6], *Edwardsiella piscicida* [2,7,8], and *Aeromonas veronii* [4]. Moreover, co-infections of different bacterial pathogens have been frequently reported in recent years [9].

Generally, bacterial pathogens typically become active when water temperature exceeds 24 °C. Largemouth bass are usually susceptible to pathogenic bacteria under unfavorable conditions, such as parasitic infections, temperature extremes, and poor water quality. These unfavorable conditions can decrease the immunity of largemouth bass and, as a result, opportunistic bacteria cause outbreaks of diseases [5,10,11]. Different bacterial infections can cause various symptoms, include gill rot, ulcer/rot/hemorrhage on the body and fins, visceral hemorrhage, enteritis, and ascites [6,12,13]. It is worth noting that many bacterial pathogens can be transmitted to humans, posing potential threats to human health.

In the study, a comprehensive investigation of potential bacterial pathogens in largemouth bass was performed. Diseased specimens were collected for bacterial isolation, and the isolates were identified and characterized by gene sequencing, morphological, and physiological–biochemical tests. Additionally, antibiotic sensitivity testing was performed to screen effective antibacterial agents. Our data will enrich the potential bacterial diseases information of largemouth bass and promote the sustainable and healthy development of the largemouth bass industry.

## 2. Materials and Methods

### 2.1. Sample Collection

Twelve areas were selected for the potential bacterial pathogen investigation, including Tongliang, Changshou, Liangping, Wanzhou, Hechuan, Yongchuan, Zhongxian, Xiushan, Fuling, Fengdu, Qijiang, and Shizhu. Monthly surveys were conducted from April to October 2024. The total number of samples for analysis was 1512. Clinical examinations were performed on diseased specimens, with characteristic symptoms being photographically documented. The diseased largemouth bass with typical clinical signs were immediately placed in oxygenated plastic bags containing original pond water and transported to the laboratory within 4 h. All animal experiments were performed in strict compliance with the ethical guidelines outlined in the Guide for the Care and Use of Laboratory Animals, following protocols approved by the Institutional Animal Care and Use Committee of Southwest University (Approval No. SWU-AF-2024-003).

### 2.2. Isolation and Purification of Bacteria

Initially, each collected largemouth bass specimen underwent thorough external examination. Visible lesions on body surfaces, gills, and fins were photographically documented before sampling. Using sterile technique, surface lesions were sampled with a disposable inoculation loop and streaked onto Luria–Bertani (LB) agar plates following standard quadrant streaking methodology. Following external examination, specimens were transferred to a Class II biological safety cabinet for aseptic dissection. Internal organs (liver, kidney, spleen) demonstrating macroscopic pathology, along with any present ascitic fluid, were systematically examined. Tissue samples from affected organs and ascites were collected using sterile instruments and inoculated onto fresh LB agar plates. All microbiological procedures were performed under strict aseptic conditions to prevent cross-contamination.

Following incubation at 28 °C for 24 h, distinct bacterial colonies were selected from LB agar plates based on morphological characteristics. Primary isolates were subjected to three successive rounds of purification by streaking onto fresh LB agar plates. During each purification cycle, colony morphology (including size, shape, color, margin, and elevation) was carefully documented. Following the final purification step, well-isolated colonies were aseptically transferred to cryopreservation tubes containing 20% (*v*/*v*) glycerol solution. The bacterial suspensions were then stored at −80 °C in an ultralow-temperature freezer for long-term preservation and subsequent experimental use. All purification procedures were performed under sterile conditions to prevent contamination.

### 2.3. DNA Sequencing

Genomic DNA was extracted from bacterial cultures using a bacterial genomic DNA extraction kit (Tiangen Biochemical Technology Co., Ltd., Beijing, China) according to the manufacturer’s protocol. PCR amplification was performed using a 2 × rapid taq master mix kit (Vazyme Biotechnology Co., Ltd., Nanjing, China) and specific primer sets targeting the 16S rRNA gene (forward: 5′-GAGAGTTTGATCCTGGCTCAG-3′; reverse: 5′-GGTTACCTTGTTACGACTT-3′) and the gyrB gene (forward: GAAGTCATCATGACCGTTCTGCAYGCNGGNGGNAARTTYGA; reverse: AGCAGGGTACGGATGTGCGAGCCRTCNACRTCNGCRTCNGTCAT). The thermal cycling conditions consisted of an initial denaturation at 95 °C for 5 min, followed by 30 cycles of denaturation (95 °C for 30 s), annealing (55 °C for 30 s), and extension (72 °C for 90 s). Amplified products were purified and sequenced by TsingKe Biotechnology Co., Ltd. (Wuhan, China).

To improve the accuracy of species identification, the isolated strains were identified by conjoint analysis of 16S rRNA and gyrB gene sequences. Briefly, the 16S rRNA and gyrB gene fragments were analyzed using BLAST against the NCBI nucleotide database (https://blast.ncbi.nlm.nih.gov/Blast.cgi?PROGRAM=blastn&PAGE_TYPE=BlastSearch&LINK_LOC=blasthome, accessed on 17 June 2025). Following alignments, the sequences with high homology to the subject strains were downloaded. For phylogenetic analysis, the 16S rRNA and gyrB gene sequences were aligned and adjusted to allow maximum alignment using the Mega-X software. The effective sequences of 16S rRNA and gyrB genes were stitched into a new sequence, and then used for species identification.

### 2.4. Transmission Electron Microscope (TEM) Observation

For ultrastructural characterization of bacterial morphology, bacterial cultures were processed as follows: logarithmic-phase cultures were harvested by centrifugation at 3000× *g* for 3 min. Cell pellets were washed three times with 0.9% (*w*/*v*) NaCl solution and then fixed in 2.5% (*v*/*v*) glutaraldehyde at 4 °C for 12 h. The fixed samples were applied to Formvar-coated copper grids (300 mesh) and allowed to adsorb for 5 min. Excess liquid was removed with filter paper, followed by negative staining with 3% (*w*/*v*) phosphotungstic acid (pH 7.2) for 90 s. Samples were examined using a JEM-1400 TEM (JEOL Ltd., Tokyo, Japan) operating at 80 kV. Digital images were acquired using a Gatan Orius SC1000 CCD camera system (Gatan, Inc., Pleasanton, CA, USA).

### 2.5. Gram Staining

Gram staining was performed using a commercial staining kit (Solarbio, Beijing, China) according to standardized microbiological protocols. Bacterial suspensions in logarithmic growth phase were prepared, and then 10 μL aliquots were spread on ethanol-cleaned microscope slides, followed by air-drying at room temperature (25 ± 1 °C). The air-dried smears were initially stained with crystal violet–ammonium oxalate solution for 90 ± 30 s, then gently rinsed with distilled water. Gram’s iodine mordant was applied for 60 s to fix the primary stain, after which slides were again rinsed with distilled water. Decolorization was performed using 95% (*v*/*v*) ethanol for 25 ± 5 s and immediately terminated by water rinsing. For counterstaining, safranin solution was applied for 150 ± 30 s before a final water rinse. Excess liquid was carefully removed by blotting with absorbent paper. The slides were either air-dried or examined immediately under oil immersion (100× objective) using an Olympus BX53 light microscope (Olympus, Tokyo, Japan). Gram-positive bacteria retained the crystal violet–iodine complex, appearing deep purple, while Gram-negative organisms were decolorized and counterstained pink-red.

### 2.6. Capsule Staining

The capsule staining was performed using a commercial staining kit (Solarbio, Beijing, China) according to the manufacturer’s protocol with minor modifications. Briefly, logarithmic-phase bacterial suspensions were prepared in sterile phosphate-buffered saline (PBS, pH 7.4). A 10 μL aliquot of bacterial suspension was aseptically transferred onto a pre-cleaned microscope slide and air-dried at room temperature (25 ± 1 °C). The dried smears were flooded with 1% (*w*/*v*) crystal violet solution and allowed to stain for 6 ± 1 min. Excess stain was carefully removed by rinsing with 20% (*w*/*v*) copper sulfate solution (3×, 10 s each). Slides were then mounted with cedarwood oil (Sigma-Aldrich, Shanghai, China) and immediately examined under an Olympus BX53 light microscope equipped with a 100× oil immersion objective. Bacterial cells stained purple while capsules remained unstained or exhibited light purple halos.

### 2.7. Motility Detection

Bacterial motility was assessed using stab inoculation technique in semi-solid LB medium. Logarithmic-phase cultures (OD_600_ = 0.4–0.6) were prepared by incubating bacterial suspensions in LB broth at 28 °C with shaking at 180 rpm. Using a flame-sterilized inoculation needle, bacterial suspension was aseptically collected and vertically stabbed to a depth of 3 cm into sterile semi-solid LB agar contained in 15 mL tubes. The inoculated tubes were incubated at 28 °C under static conditions. Motility was determined by examining the migration pattern from the central stab line, where motile strains demonstrated diffuse growth radiating from the inoculation line, while non-motile strains showed growth restricted to the stab line.

### 2.8. Hemolytic Test

Hemolytic activity was evaluated using sheep blood agar plates prepared by aseptically adding 10% (*v*/*v*) defibrinated sheep blood (Solarbio, China) to sterilized blood agar base medium cooled to 50 ± 2 °C. The mixture was gently homogenized and dispensed into sterile Petri dishes (15 mL per plate). Bacterial suspensions in mid-logarithmic growth phase (OD_600_ = 0.4–0.6) were spot-inoculated (5 μL) onto the prepared plates in triplicate. Following incubation at 28 °C for 18 h under aerobic conditions, hemolytic patterns were assessed by examining zones of clearing around colonies: complete (β-hemolysis), partial (α-hemolysis), or absent (γ-hemolysis).

### 2.9. Biochemical Identification

Biochemical identification was carried out using bacterial microbial biochemical identification kits (HaiBo Biotechnology Co., Ltd., Qingdao, China). The tests included Methyl Red (MR) and Voges–Proskauer (VP) assays to assess sugar metabolism pathways, o-nitrophenyl-β-D-galactopyranoside (ONPG) testing for β-galactosidase activity, urease (Ur) and gelatin hydrolysis (GH) assays, as well as amino acid metabolism tests (arginine dihydrolase (AD), ornithine decarboxylase (OD), and lysine decarboxylase (LD)). Additional evaluations included oxidase (Ox) activity and carbohydrate utilization tests (mannitol (Mal), mannose (Mae), lactose (La), cellobiose (Ce), arabinose (Ar), and sucrose (Su)). For specific steps, the instruction manual of this set of biochemical identification tubes was referred to.

### 2.10. Antibiotic Sensitivity Testing

Antibiotic sensitivity testing was performed following the standard Kirby–Bauer disk diffusion method and conducted using 11 aquaculture-approved antibiotics authorized by the Ministry of Agriculture and Rural Affairs of China. Tested antibiotics included: thiamphenicol (THI; 30 μg/disc), florfenicol (FLO; 30 μg/disc), flumequine (FLU; 30 μg/disc), enrofloxacin (ENR; 5 μg/disc), doxycycline hyclate (DOH; 30 μg/disc), neomycin sulfate (NES; 30 μg/disc), sulfamonomethoxine sodium (SUS; 30 μg/disc), a premix of vitamin C magnesium phosphate and ciprofloxacin hydrochloride (VcMP-CiH; 50/5 μg/disc), compound sulfadiazine (Sud-Tri; sulfadiazine/trimethoprim 50/6.25 μg/disc), compound sulfadimidine (Sum-Tri; sulfadimidine/trimethoprim 25/5 μg/disc), and compound sulfamethoxazole (Suo-Tri: sulfamethoxazole/trimethoprim 25/5 μg/disc). Antimicrobial discs were prepared by punching 6 mm diameter circles from sterile filter paper. Antibiotic solutions were prepared with water, methanol, or dimethyl sulfoxide and then filtrated through 0.22-micron filters. Each disc was dropped with 8 μL of antibiotic solution, air-dried under laminar flow, and stored at 4 °C or −20 °C. For testing, logarithmic-phase cultures of the bacterial isolates were evenly spread on LB agar plates, followed by application of antibiotic discs. The inhibition zone is defined as the diameter of the zone of bacterial growth inhibition around the disc. The zone of bacterial growth inhibition was calculated as the mean diameter from three replicates.

## 3. Results and Discussion

As one of the most important freshwater fishes, largemouth bass has been widely farmed around the world. It was firstly introduced into China in the 1980s and rapidly progressed to a dominant economic fish. However, increasing disease challenges pose a critical bottleneck to the sustainable development of largemouth bass farming [13]. Among these challenges, bacterial diseases stand out as the most prevalent and detrimental category, characterized by abrupt onset, rapid transmission, and high mortality rates [4,14]. In the study, a comprehensive potential bacterial pathogens investigation was performed in 12 areas. The results will provide critical data to support evidence-based disease management strategies and promote the sustainable and healthy development of the largemouth bass industry.

### 3.1. Clinical Signs of the Diseased Largemouth Bass

Water temperature is an important environmental factor affecting the occurrence and severity of bacterial diseases [15]. When the water temperature exceeds 24 °C, the activity of bacteria in the water significantly increases, and their reproduction rate also accelerates greatly, making fish more vulnerable to bacterial infections. In addition, high temperatures could also deteriorate the aquaculture environment, such as decreases in oxygen content and accumulation of harmful substances. These changes weakened the immunity of largemouth bass, making them more likely to be infected by bacterial diseases [16]. Based on above, monthly surveys of potential bacterial pathogens were conducted from April to October 2024.

**Figure 1:**
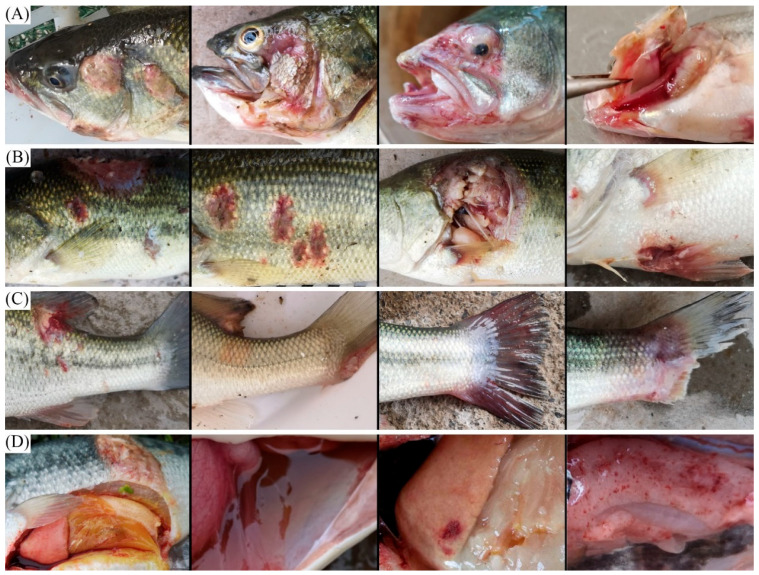
Clinical symptoms of the diseased largemouth bass. (**A**) Cephalic region: exophthalmos, opercular and oral ulceration, and gill rot; (**B**) Body surface: multiple cutaneous ulcers, particularly severe on the abdominal surface; (**C**) Fins: basal ulceration and partial fin erosion; (**D**) Visceral organs: abdominal ascites accumulation and hepatic pallor with petechial hemorrhages.

**Figure 2:**
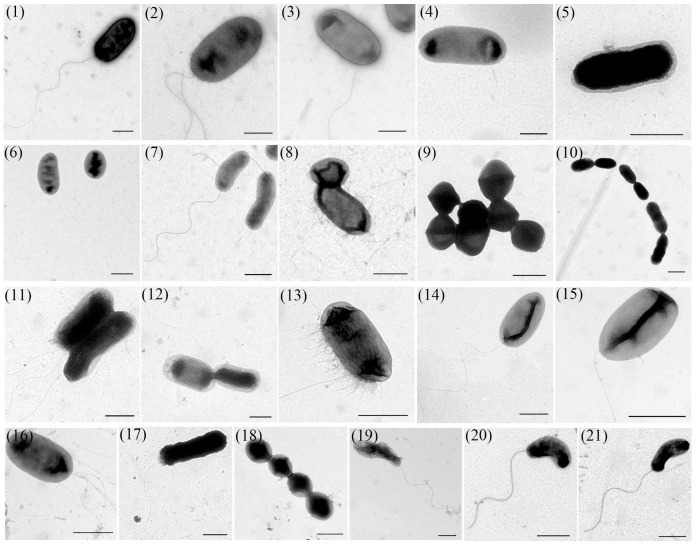
Representative TEM images of the 21 bacterial strains (Scale bars: 1 μm). (1) *Aeromonas caviae*; (2) *Aeromonas dhakensis*; (3) *Aeromonas hydrophila*; (4) *Aeromonas jandaei*; (5) *Aeromonas salmonicida*; (6) *Aeromonas veronii*; (7) *Chryseobacterium indologenes*; (8) *Edwardsiella piscicida*; (9) *Enterococcus faecalis*; (10) *Lactococcus garvieae*; (11) *Photobacterium damselae*; (12) *Plesiomonas shigelloides*; (13) *Proteus vulgaris*; (14) *Pseudomonas aeruginosa*; (15) *Pseudomonas parafulva*; (16) *Pseudomonas putida*; (17) *Shewanella xiamenensis*; (18) *Streptococcus agalactiae*; (19) *Vibrio alginolyticus*; (20) *Vibrio cholerae*; (21) *Vibrio parahaemolyticus*.

**Figure 3:**
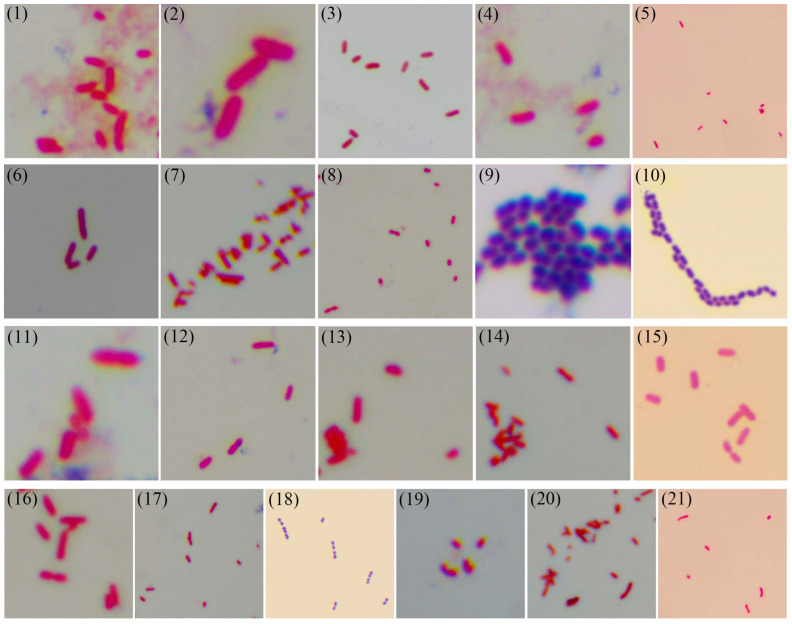
Gram staining of the 21 bacterial strains. (1) *Aeromonas caviae*; (2) *Aeromonas dhakensis*; (3) *Aeromonas hydrophila*; (4) *Aeromonas jandaei*; (5) *Aeromonas salmonicida*; (6) *Aeromonas veronii*; (7) *Chryseobacterium indologenes*; (8) *Edwardsiella piscicida*; (9) *Enterococcus faecalis*; (10) *Lactococcus garvieae*; (11) *Photobacterium damselae*; (12) *Plesiomonas shigelloides*; (13) *Proteus vulgaris*; (14) *Pseudomonas aeruginosa*; (15) *Pseudomonas parafulva*; (16) *Pseudomonas putida*; (17) *Shewanella xiamenensis*; (18) *Streptococcus agalactiae*; (19) *Vibrio alginolyticus*; (20) *Vibrio cholerae*; (21) *Vibrio parahaemolyticus*.

**Figure 4:**
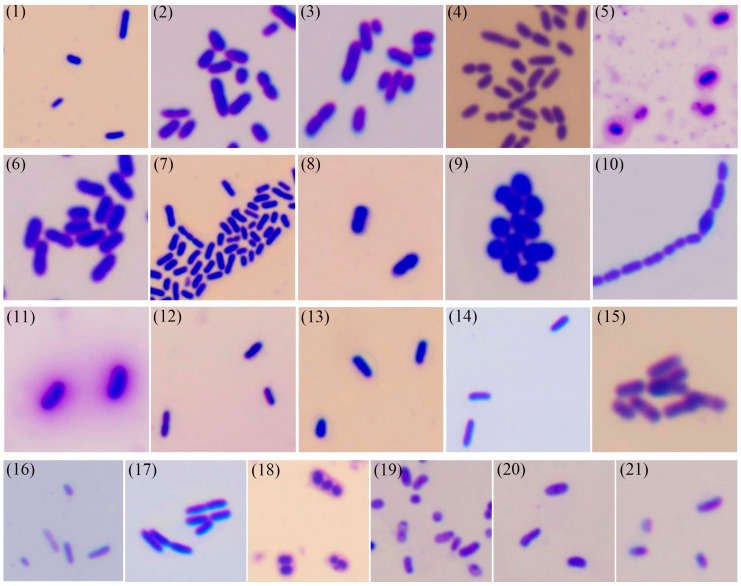
Capsule staining of the 21 bacterial strains. (1) *Aeromonas caviae*; (2) *Aeromonas dhakensis*; (3) *Aeromonas hydrophila*; (4) *Aeromonas jandaei*; (5) *Aeromonas salmonicida*; (6) *Aeromonas veronii*; (7) *Chryseobacterium indologenes*; (8) *Edwardsiella piscicida*; (9) *Enterococcus faecalis*; (10) *Lactococcus garvieae*; (11) *Photobacterium damselae*; (12) *Plesiomonas shigelloides*; (13) *Proteus vulgaris*; (14) *Pseudomonas aeruginosa*; (15) *Pseudomonas parafulva*; (16) *Pseudomonas putida*; (17) *Shewanella xiamenensis*; (18) *Streptococcus agalactiae*; (19) *Vibrio alginolyticus*; (20) *Vibrio cholerae*; (21) *Vibrio parahaemolyticus*.

Diseased largemouth bass exhibited characteristic clinical symptoms across multiple regions: (1) cephalic region: exophthalmos, opercular and oral ulceration, and gill rot (Figure 1A); (2) integumentary system: multiple cutaneous ulcers, particularly severe on the abdominal surface (Figure 1B); (3) fins: basal ulceration and partial fin erosion (Figure 1C); (4) visceral organs: abdominal ascites accumulation and hepatic pallor with petechial hemorrhages (Figure 1D).

Similar pathological presentations have been documented in other studies of bacterial infections [2,17,18]. Meanwhile, some studies have shown that largemouth bass infected with *Plesiomonas shigelloides* exhibited a series of symptoms, including anorexia, ulceration at the base of fish fins, hemorrhage on body surface, and ascites [19]. In addition, *Aeromonas veronii* has emerged as a significant pathogen, causing opercular and oral ulceration, fin erosion, exophthalmos, hepatic atrophy, and splenomegaly [4,14]. *Edwardsiella piscicida* infections manifest as abdominal distension, ascites, hepatic pallor, and cutaneous ulceration [2]. These overlapping clinical symptoms underscore the necessity for comprehensive diagnostic approaches for accurate pathogen identification.

### 3.2. Species Identification

Precise identification of bacterial pathogens necessitates molecular methods, principally through sequencing of conserved housekeeping genes including 16S rRNA, gyrase B (gyrB), and RNA polymerase beta subunit (rpoD) [20]. While 16S rRNA serves as a standard housekeeping gene in bacterial taxonomy, its discriminatory power is often insufficient for closely related species. The *gyrB* gene provides higher phylogenetic resolution for such taxonomic distinctions [21,22]. Therefore, in order to improve the accuracy of the results, a method of jointly analyzing the sequences of the 16S rRNA gene and the gyrB gene was performed to identify the isolated strains.

The 16S rRNA and gyrB gene fragments were respectively analyzed using BLAST against the NCBI nucleotide database (Supplementary Tables S1 and S2). The effective sequences of 16S rRNA and gyrB genes were stitched into a new sequence and then used for species identification. Following isolation and identification, a total of 21 potential bacterial pathogens (numbered strain 1 to 21, respectively) were successfully identified to species level (Table 1). Results showed that, among the 21 isolated strains, the genus *Aeromonas* accounted for the highest proportion (67.14%), among which the frequency of *Aeromonas veronii* was 24.60%.

Previous studies have isolated various *Aeromonas* bacteria from diseased largemouth bass, such as *Aeromonas hydrophila* [14] and *Aeromonas veronii* [4]. Meanwhile, our laboratory reported a case of largemouth bass co-infected with *Aeromonas dhakensis* and *Chryseobacterium indologenes* [9], which further expanded the known range of potential pathogens. Additionally, we also isolated *Vibrio parahaemolyticus*, *Vibrio alginolyticus*, and *Vibrio cholerae*. Several studies have documented that some *Vibrio* species are important pathogens, including *Vibrio parahaemolyticus* [23] and *Vibrio alginolyticus* [24], and relevant research has focused on their pathogenicity and the host immune response. Other significant bacterial pathogens identified in largemouth bass include *Edwardsiella piscicida* [8], *Streptococcus agalactiae*, and *Lactococcus garvieae* [25]. The synergistic effects of co-infections, such as between *Aeromonas hydrophila* and *Pseudomonas aeruginosa* [26], and comprehensive disease management strategies incorporating *Photobacterium damselae* infections [13] have also been investigated.

### 3.3. TEM Observation

TEM analysis revealed considerable morphological diversity among these isolates (Figure 2). Bacterial dimensions ranged from 0.5 to 3 μm in length, exhibiting three predominant shapes: rod-shaped (strains 1–8, 11–17), spherical (strains 9, 10, 18), and curved forms (strains 19–21). Flagellar structures were observed in strains 1–4, 6–8, 11–17, 19–21, with variations in both number and insertion sites (peritrichous or polar flagellation). The spherical bacterium demonstrated uniform cellular diameter, while rod-shaped isolates showed substantial length polymorphism, ranging from relatively short forms to elongated morphotypes.

Bacterial morphology represents an evolutionary adaptation influencing critical physiological processes, including nutrient uptake, motility, and environmental stress resistance [27]. The observed morphological consistency across generations permits preliminary taxonomic assessment. For instance, the cells of strain 18 are spherical and connected linearly, arranged in chains, and have no flagella, which is consistent with the typical morphology of *Streptococcus agalactiae* [28]. Among aquatic pathogens, rod-shaped bacteria predominated, including members of *Aeromonas*, *Vibrio*, *Edwardsiella*, and *Pseudomonas* genera [29]. *Aeromonas* species (e.g., *Aeromonas hydrophila, Aeromonas veronii*) typically measure 0.3–1.0 μm × 1.0–3.5 μm and cause ulcerative dermal lesions and septicemia in aquatic hosts. *Vibrio* spp., including *Vibrio parahaemolyticus* and *Vibrio alginolyticus*, exhibit characteristic curved rods (0.5–0.8 μm × 1.4–2.6 μm) and induce vibriosis manifesting as anorexia, skin ulceration, and pigmentation changes [10,30].

### 3.4. Gram Staining

Gram staining of the 21 bacterial isolates was performed. As shown in Figure 3 and Table 2 (“+” indicates Gram-positive reaction, while “−” indicates Gram-negative reaction), three isolates (strains 9, 10, 18) demonstrated Gram-positive characteristics, while the remaining strains were Gram-negative.

This differential staining reflects fundamental differences in cell wall architecture between Gram-positive and Gram-negative bacteria. Gram-positive cell walls feature a thick (20–80 nm) peptidoglycan layer containing teichoic acids and phosphoglucuronic acid, whereas Gram-negative organisms possess a thinner (2–7 nm) peptidoglycan layer sandwiched between the cytoplasmic membrane and an outer membrane rich in lipopolysaccharides [31]. These structural differences account for the distinct staining patterns: Gram-positive bacteria retain crystal violet–iodine complexes due to ethanol-induced peptidoglycan pore constriction, while Gram-negative bacteria lose these complexes through increased permeability from lipid dissolution [32]. The observed Gram-positive isolates may exhibit greater susceptibility to cell wall-targeting antibiotics compared to their Gram-negative counterparts, which benefit from additional outer membrane protection [28].

### 3.5. Capsule Staining

Capsule production of the 21 bacterial isolates was assessed. As shown in Figure 4 and Table 2 (“Y” indicates the presence of capsule, while “N” indicates the absence of capsule), only three isolates (strains 5, 11, 18) demonstrated capsule formation, representing 14.3% of the total isolates, while the remaining 18 isolates were non-capsulated.

Bacterial capsules are extracellular mucoid structures primarily composed of polysaccharides, and some consist of polypeptides (e.g., *Bacillus anthracis* capsule; [11]). These structures occur in both Gram-positive and Gram-negative species, including clinically significant pathogens such as *Streptococcus pneumoniae*, *Haemophilus influenzae*, and *Klebsiella pneumoniae* [33]. Capsules mediate critical virulence functions through antiphagocytic activity and environmental resistance [34,35], with distinct membrane attachment mechanisms in Gram-positive (phospholipid/lipid A linkage) versus Gram-negative (peptidoglycan covalent binding) bacteria [11]. The observed capsule production in strains 5, 11, and 18 suggests potentially enhanced pathogenicity compared to non-capsulated isolates. Emerging evidence suggests capsule-mediated antibiotic resistance mechanisms may influence treatment efficacy in aquaculture settings [36,37], highlighting the practical value of capsule detection for informing targeted disease management strategies.

### 3.6. Motility Detection

**Figure 5:**
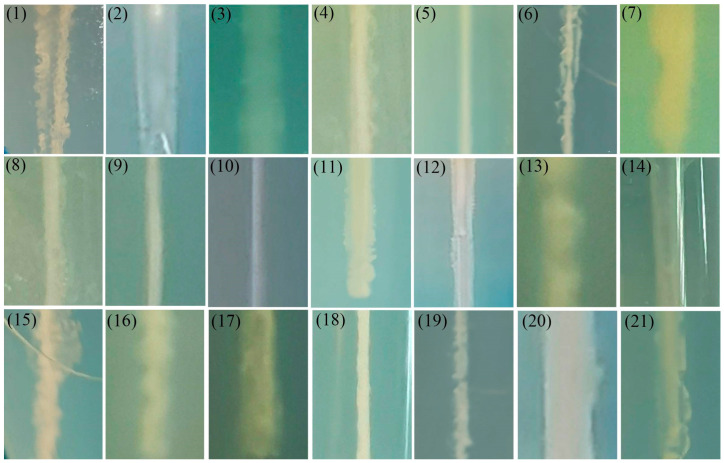
Bacterial motility detection of the 21 bacterial strains. (1) *Aeromonas caviae*; (2) *Aeromonas dhakensis*; (3) *Aeromonas hydrophila*; (4) *Aeromonas jandaei*; (5) *Aeromonas salmonicida*; (6) *Aeromonas veronii*; (7) *Chryseobacterium indologenes*; (8) *Edwardsiella piscicida*; (9) *Enterococcus faecalis*; (10) *Lactococcus garvieae*; (11) *Photobacterium damselae*; (12) *Plesiomonas shigelloides*; (13) *Proteus vulgaris*; (14) *Pseudomonas aeruginosa*; (15) *Pseudomonas parafulva*; (16) *Pseudomonas putida*; (17) *Shewanella xiamenensis*; (18) *Streptococcus agalactiae*; (19) *Vibrio alginolyticus*; (20) *Vibrio cholerae*; (21) *Vibrio parahaemolyticus*.

The puncture assay revealed distinct motility patterns among the 21 bacterial isolates (Figure 5). There was no diffusion of the inoculation lines after the puncture of strains 5, 9, 10, and 18, indicating that these strains were non-motile; while the inoculation lines of the remaining strains showed diffusion, which meant that they had active motility.

Bacterial motility, mediated primarily by flagellar and pili structures, encompasses diverse movement modalities including swimming, swarming, twitching, and gliding [38]. This capability enables microorganisms to navigate chemical gradients, colonize optimal niches, and evade hostile environments [39]. Pathogens such as *Salmonella* spp. and *Vibrio cholerae* exemplify how flagellar motility facilitates tissue penetration and colonization [40], while *Helicobacter pylori* demonstrates flagellar-dependent gastric mucus penetration [41]. Motility significantly contributes to biofilm formation and virulence regulation. *Pseudomonas aeruginosa* coordinates flagellar movement with exotoxin production [42], and biofilm-associated motility enhances both environmental persistence and antimicrobial resistance [43].

### 3.7. Hemolytic Test

**Figure 6:**
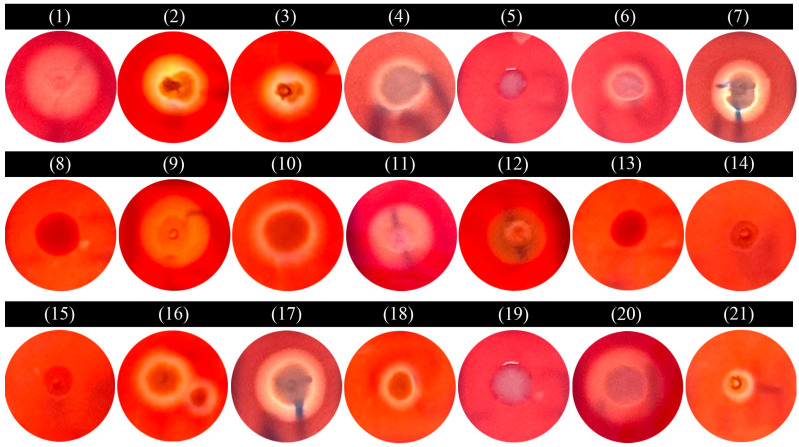
Hemolytic test of the 21 bacterial strains. (1) *Aeromonas caviae*; (2) *Aeromonas dhakensis*; (3) *Aeromonas hydrophila*; (4) *Aeromonas jandaei*; (5) *Aeromonas salmonicida*; (6) *Aeromonas veronii*; (7) *Chryseobacterium indologenes*; (8) *Edwardsiella piscicida*; (9) *Enterococcus faecalis*; (10) *Lactococcus garvieae*; (11) *Photobacterium damselae*; (12) *Plesiomonas shigelloides*; (13) *Proteus vulgaris*; (14) *Pseudomonas aeruginosa*; (15) *Pseudomonas parafulva*; (16) *Pseudomonas putida*; (17) *Shewanella xiamenensis*; (18) *Streptococcus agalactiae*; (19) *Vibrio alginolyticus*; (20) *Vibrio cholerae*; (21) *Vibrio parahaemolyticus*.

Hemolytic patterns of the 21 bacterial isolates were characterized using blood agar assays (Figure 6 and Table 2). Distinct hemolytic phenotypes were observed: isolates 1–4, 6, 7, 9–11, 16–18, and 21 exhibited β-hemolysis, demonstrating complete erythrocyte lysis with wide and transparent zones; isolates 5, 8, 13–15, and 19 showed γ-hemolysis (non-hemolytic); while isolate 12 displayed α-hemolysis with partial erythrocyte lysis evidenced by narrow and greenish-brown zones.

The predominance of β-hemolytic isolates (66.7%) suggests significant hemolysin production, potentially enhancing pathogenicity through erythrocyte destruction and subsequent physiological disruption during infection. In contrast, γ-hemolytic isolates likely employ alternative virulence mechanisms independent of hemolysis. The intermediate α-hemolytic phenotype may indicate distinct hemolysin mechanisms [44,45]. Hemolysins represent a critical virulence factor class, mediating membrane disruption through pore-forming activity [46]. These toxins facilitate bacterial pathogenesis through multiple mechanisms: immune evasion *(Staphylococcus aureus* α-hemolysin; [47], tissue barrier penetration (*Streptococcus pyogenes* streptolysin O; [48], and biofilm maintenance (*Pseudomonas aeruginosa* hemolysins; [49].

### 3.8. Biochemical Identification

Physiological and biochemical characteristics of the 21 bacterial isolates were comprehensively analyzed (Table 2). The metabolic profiles exhibited significant diversity among isolates, as indicated by positive (“+”) or negative (“−”) reactions in each test. These physiological and biochemical characteristics provide valuable insights for bacterial characteristics. For example, the MR-VP tests differentiated sugar fermentation pathways, while oxidase activity helped distinguish bacterial genera. Variations in carbohydrate utilization patterns reflected niche-specific adaptations, and salt tolerance indicated osmotic stress response capabilities [17,50]. However, the interpretation of these results has limitations. Individual tests may yield variable outcomes, and the complex interactions between metabolic pathways can complicate comprehensive analysis. The current data can serve as a foundation for understanding the functional diversity of these bacterial isolates.

Integration of DNA identification with morphological characterization and physiological–biochemical tests provided a multidimensional characterization of these isolates. Gram differentiation revealed fundamental structural differences between Gram-positive and Gram-negative isolates, with implications for antibiotic susceptibility patterns. Physiological tests demonstrated diverse metabolic capabilities reflecting environmental adaptations, particularly in carbohydrate and protein utilization pathways. While these analyses offer substantial insights into bacterial classification and potential virulence mechanisms, they have limitations in predicting complex ecological interactions and antibiotic resistance patterns. Therefore, we conducted additional antimicrobial susceptibility testing to directly assess therapeutic options, providing critical data for clinical treatment and aquaculture disease management. The combined results establish a foundation for understanding pathogen diversity and developing targeted control measures for largemouth bass aquaculture.

### 3.9. Antibiotic Sensitivity of the Isolated Strains

In the process of aquaculture, the indiscriminate application of antibiotics creates selective pressures that favor the proliferation of resistant bacterial strains while eliminating susceptible populations. This ecological shift not only compromises treatment efficacy but also facilitates the dissemination of resistance genes through trophic transfer, posing substantial risks to both public health and ecosystem stability [51,52]. In the study, we evaluated the susceptibility profiles of the isolated strains against 11 clinically approved antibiotics for aquaculture. These agents have been authorized by the Ministry of Agriculture and Rural Affairs of China and widely used in aquaculture [53,54].

As shown in Table 1, florfenicol (FLO) exhibited extremely excellent antibacterial effects. It formed large inhibition zones (>30 mm) for numerous bacterial species such as *Vibrio parahaemolyticus*, *Aeromonas salmonicida*, *Vibrio alginolyticus*, *Streptococcus agalactiae*, *Shewanella xiamenensis*, *Aeromonas jandaei*, and *Photobacterium damselae*. Meanwhile, flumequine (FLU), enrofloxacin (ENR), and VcMP-CiH (vitamin C magnesium phosphate:ciprofloxacin hydrochloride = 10:1) also showed certain antibacterial activities against various bacteria, which was consistent with the research results of previous researchers [55]. In contrast, the antibacterial performance of SUS, Sud-Tri, Sum-Tri, and Suo-Tri was poor, indicating that the bacteria had low sensitivity to these drugs. Especially for SUS, there were no inhibition zones for all bacteria except *Aeromonas jandaei*. According to the results, *Plesiomonas shigelloides* and *Pseudomonas putida* showed resistance to most of the tested antibiotics, which was also consistent with the previous research results [56].

The resistance patterns exhibited differences not only between genera but also within the same genus. Distinct susceptibility characteristics could be clearly observed among different species of *Vibrio*, *Aeromonas*, and *Enterococcus*. Even among the isolates of the same genus, *Aeromonas*, the responses to the same antibiotics varied from one isolate to another [57]. For example, in response to florfenicol (FLO), the average diameter of the inhibition zone of *Aeromonas dhakensis* was 8 mm, while that of *Aeromonas jandaei* was 32 mm. In response to thiamphenicol (THI), there was no inhibition zone for *Aeromonas veronii*, while the average diameter of the inhibition zone of *Aeromonas jandaei* was 38 mm, showing a significant difference. These resistance patterns emerged through environmental selection pressures in aquaculture systems, where prolonged antibiotic exposure enriched resistant subpopulations through selective elimination of susceptible strains. These findings carried significant implications for aquaculture management and clinical practice. Targeted antibiotic selection based on resistance profiles could optimize therapeutic effects and mitigate resistance development. These data provided an evidence-based framework for antibiotic application in aquatic animal health management.

## 4. Conclusions

In the study, a comprehensive investigation on potential bacterial pathogens affecting largemouth bass is performed. A total of 21 different potential bacterial pathogens are isolated and identified by gene sequencing, morphological characterization, and physiological–biochemical tests. Most bacterial strains are relatively sensitive to florfenicol, enrofloxacin, and VcMP-CiH, making them first-choice drugs for disease control. These data will enrich the potential bacterial diseases information and promote the healthy development of the largemouth bass industry.

## Figures and Tables

**Table 1:** Bacterial species, occurrence frequency, and antibiotic sensitivity of the isolated strains.

S. No.	Species	Frequency (%)	Mean Inhibition Zone Diameter (mm)										
			THI	FLO	FLU	ENR	DOH	NES	SUS	VcMP-CiH	Sud-Tri	Sum-Tri	Suo-Tri
1	*Aeromonas caviae*	3.76	-	12	34	32	12	16	-	24	-	-	-
2	*Aeromonas dhakensis*	4.63	-	8	38	36	12	22	-	30	-	-	-
3	*Aeromonas hydrophila*	20.98	-	9	32	34	8	21	-	24	-	-	-
4	*Aeromonas jandaei*	4.05	38	32	38	40	36	22	14	42	28	28	26
5	*Aeromonas salmonicida*	9.12	26	32	24	22	12	15	-	27	22	23	22
6	*Aeromonas veronii*	24.60	-	12	18	22	14	16	-	28	-	-	-
7	*Chryseobacterium indologenes*	1.88	14	26	30	25	24	10	-	20	22	24	28
8	*Edwardsiella piscicida*	6.51	-	-	26	22	-	12	-	30	-	-	-
9	*Enterococcus faecalis*	0.87	-	26	-	18	-	-	-	22	33	26	24
10	*Lactococcus garvieae*	1.01	16	19	9	16	9	9	-	15	-	-	-
11	*Photobacterium damselae*	2.75	-	40	-	22	-	16	-	22	33	32	32
12	*Plesiomonas shigelloides*	3.47	-	14	8	-	-	-	-	-	-	-	-
13	*Proteus vulgaris*	1.59	9	20	28	26	14	16	-	34	24	20	22
14	*Pseudomonas aeruginosa*	2.75	11	10	14	22	9	8	-	30	13	-	10
15	*Pseudomonas parafulva*	0.72	11	22	14	19	14	17	-	25	-	-	-
16	*Pseudomonas putida*	1.74	-	8	-	-	-	18	-	-	-	-	-
17	*Shewanella xiamenensis*	0.72	30	41	11	9	19	22	-	-	29	28	28
18	*Streptococcus agalactiae*	3.04	22	32	-	22	22	-	-	21	24	14	-
19	*Vibrio alginolyticus*	2.03	26	32	25	26	14	16	-	24	18	14	18
20	*Vibrio cholerae*	1.30	26	25	28	24	16	14	-	28	25	23	24
21	*Vibrio parahaemolyticus*	2.46	25	32	24	22	12	16	-	24	21	18	24

**Notes:** S. No.: Strain number; THI: Thiamphenicol; FLO: Florfenicol; FLU: Flumequine; ENR: Enrofloxacin; DOH: Doxycycline hydrochloride; NES: Neomycin sulfate; SUS: Sulfamonomethoxine sodium; VcMP-CiH: (Vitamin C magnesium phosphate: Ciprofloxacin hydrochloride = 10:1); Sud-Tri: (Sulfadiazine: Trimethoprim = 8:1); Sum-Tri: (Sulfamethazine: Trimethoprim = 5:1); Suo-Tri: (Sulfamethoxazole: Trimethoprim = 5:1); “-” = non-bacteriostatic function.

**Table 2:** Morphological characterization and physiological–biochemical tests of the 21 bacterial strains.

S. No.	Mor	Fla	Cap	GR	Hem	MR-VP	ONPG	Ur	GH	AD	OD	LD	Ox	NaCl Tryptone Water					1% NaCl					
														0%	3%	6%	8%	10%	Mal	Mae	La	Ce	Ar	Su
1	R	Y	N	−	β	+	+	−	+	+	+	+	+	+	+	+	+	−	+	+	+	+	+	+
2	R	Y	N	−	β	+	+	−	+	+	+	+	+	+	+	+	+	−	+	+	+	+	+	+
3	R	Y	N	−	β	+	+	−	+	+	+	+	+	+	+	+	+	−	+	+	+	+	+	+
4	R	Y	N	−	β	+	+	−	+	+	+	+	−	+	+	+	−	−	+	+	−	−	−	+
5	R	N	Y	−	γ	+	+	−	+	+	−	−	+	+	+	+	+	−	+	+	+	+	+	+
6	R	Y	N	−	β	+	+	−	+	+	+	+	+	+	+	+	+	−	+	+	+	+	+	+
7	R	Y	N	−	β	+	+	+	−	+	−	+	−	+	+	+	+	+	+	+	−	+	+	+
8	R	Y	N	−	γ	−	−	−	−	−	+	+	−	+	+	−	−	−	−	+	−	−	−	−
9	C	N	N	+	β	+	+	−	−	+	−	−	−	+	+	+	−	−	+	+	+	+	−	+
10	C	N	N	+	β	+	+	−	+	+	+	+	−	+	+	+	+	+	−	+	−	+	+	+
11	R	Y	Y	−	β	+	−	+	−	+	−	+	−	−	+	+	−	−	+	+	−	−	−	−
12	R	Y	N	−	α	−	+	−	−	+	+	+	−	+	+	−	−	−	−	−	+	−	−	−
13	R	Y	N	−	γ	−	+	+	+	+	−	+	+	+	+	+	+	+	+	+	+	+	−	+
14	R	Y	N	−	γ	+	+	−	+	+	+	−	+	+	+	+	+	−	+	+	−	−	−	−
15	R	Y	N	−	γ	+	+	−	−	+	−	−	−	+	+	+	−	−	+	+	+	+	+	+
16	R	Y	N	−	β	+	+	−	+	+	−	−	−	+	+	+	−	−	+	+	+	+	+	+
17	R	Y	N	−	β	−	−	−	+	+	+	+	−	+	+	−	−	−	+	+	−	+	−	+
18	C	N	Y	+	β	−	−	−	−	+	+	+	−	+	−	−	−	−	−	+	−	+	+	−
19	CR	Y	N	−	γ	+	−	−	−	−	+	+	+	−	+	+	+	+	+	+	−	+	−	+
20	CR	Y	N	−	β	+	+	−	+	−	+	+	+	+	+	+	−	−	+	+	−	−	−	+
21	CR	Y	N	−	β	−	**+**	**+**	**−**	**−**	**+**	**+**	+	−	+	+	+	−	+	+	−	+	+	−

**Notes:** S. No.: Strain number; Mor: Morphology; Fla: Flagella; Cap: Capsule; GR: Gram reaction; Hem: Hemolysis; R: Rods; CR: Curved rods; C: Cocci; Y: Yes; N: No; MR-VP: Methyl Red (MR) and Voges–Proskauer (VP); ONPG: o-nitrophenyl-β-D-galactopyranoside; Ur: Urease; GH: Gelatin hydrolysis; AD: Arginine dihydrolase; OD: Ornithine decarboxylase; LD: Lysin decarboxylase; Ox: Oxidase; Mal: Mannitol; Mae: Mannose; La: Lactose; Ce: Cellobiose; Ar: Arabinose; Su: Sucrose; *+*: Positive; *–*: Negative.

## Data Availability

The original contributions presented in this study are included in the article. Further inquiries can be directed to the corresponding authors.

## Supplementary Materials

The following supporting information can be downloaded at: https://www.mdpi.com/article/10.3390/microorganisms13061413/s1, Table S1: The BLAST results for 16S rRNA gene of the isolated strains; Table S2: The BLAST results for gyrB gene of the isolated strains.
