# Supplementary material for: A Comprehensive Investigation of Potential Bacterial Pathogens in Largemouth Bass (Micropterus salmoides)
Source: Microorganisms. 2025 Jun 17;13(6):1413. doi: 10.3390/microorganisms13061413 (PMC12196057; doi:10.3390/microorganisms13061413)
Supplement: Supplementary file 1 [file microorganisms-13-01413-s001.zip › microorganisms-3657407-supplementary.pdf]

Supplemental Information for

A comprehensive investigation of potential bacterial pathogens in largemouth bass (*Micropterus salmoides*)

Yun-Yao Tu[1,†], Qun Lu[1,†], Na Zhang[1], Jie Leng[1], Qin Yang[1], Jie Yu[1], Cheng-Ke Zhu[1,2], Tao He[1,2], Jian-Yong Hu[3], Ming-Ji Lv[1,*], Song Zhu[1,2,*]

[1]College of Fisheries, Southwest University, Chongqing 400715, China

[2]Key Laboratory of Freshwater Fish Reproduction and Development (Ministry of Education), Key Laboratory of Aquatic Science of Chongqing, Southwest University, Chongqing 400715, China

[3]Xinjiang Fisheries Research Institute, Scientific Observing and Experimental Station of Fishery Resources and Environment in Northwest China, Ministry of Agriculture, Urumqi 830000, China

†These authors contributed equally to this work.

*Corresponding authors: Ming-Ji Lv (18792830934@163.com); Song Zhu (zs20200801@swu.edu.cn)

Supplementary Table S1. The BLAST results for 16S rRNA gene of the isolated strains.

S. No.	Bacterial species	Percentage identity	Accession number of the closest match	E-value	Query coverage	Reference sequences used for phylogenetic tree construction
1	*Aeromonas caviae*	99.21%	OY324140.1	0.0	100.00%	OY324140.1, ON202886.1, ON202856.1, ON202870.1, OY324144.1, ON202867.1, KP262417.1, ON202844.1, JX872321.1, KP262415.1
2	*Aeromonas dhakensis*	100.00%	PQ656660.1	0.0	100.00%	PQ656660.1, CP023141.1, CP054854.1, CP170761.1, CP178405.1, CP026222.1, AP019195.1, AP022013.1, CP121802.1, AP021927.1
3	*Aeromonas hydrophila*	100.00%	CP016380.1	0.0	100.00%	CP016380.1, OQ625314.1, OQ625312.1, CP046954.1, MT974689.1, MG428923.1, PQ312918.1, MG428772.1, MG428738.1, CP113533.1
4	*Aeromonas jandaei*	100.00%	CP084376.1	0.0	100.00%	CP084376.1, CP077401.1, CP053882.1, MF716712.1, ON203017.1, KU525084.1, CP149571.1, OL884620.1, CP053881.1, CP102367.1
5	*Aeromonas*	100.00%	CP080042.1	0.0	100.00%	CP080042.1, NR026089.2, ON872224.1, CP060030.1,

	salmonicida					CP050187.1, NR113635.1, CP146071.1, FR853553.1, NR104935.1, CP021654.1
6	*Aeromonas veronii*	98.41%	CP077210.1	0.0	100.00%	CP077210.1, CP012504.1, CP024930.1, CP002607.1, CP033604.1, CP059396.1, CP136589.1, CP080040.1, OY731274.1, CP015448.1
7	*Chryseobacterium indologenes*	100.00%	OK398275.1	0.0	100.00%	OK398275.1, CP033828.1, CP192278.1, CP033930.1, MK534063.1, MH628236.1, NR112975.1, MT793133.1, CP192280.1, CP192274.1
8	*Edwardsiella piscicida*	99.93%	CP150944.1	0.0	100.00%	CP150944.1, CP150934.1, CP150925.1, AP039424.1, CP084433.1, CP084514.1, CP150927.1, CP116003.1, CP150938.1, CP150931.1
9	*Enterococcus faecalis*	100.00%	CP050648.1	0.0	100.00%	CP050648.1, CP160447.1, CP064406.1, CP030110.1, CP050650.1, LC461016.2, CP082267.1, CP091221.1, CP042839.1, OR775719.1
10	*Lactococcus garvieae*	100.00%	MG786384.1	0.0	100.00%	MG786384.1, KX671996.1, MK743983.1, MW041150.1, OK326725.1, LC484772.1, MT597707.1, LC484744.1, KM409697.1, MK559554.1

11	*Photobacterium damselae*	100.00%	PQ569048.1	0.0	100.00%	PQ569048.1, MN192102.1, PQ569047.1, MN258939.1, AP026780.1, MN263233.1, MZ026476.1, MK874935.1, CP113238.1, MN263227.1
12	*Plesiomonas shigelloides*	99.93%	LT575468.1	0.0	100.00%	LT575468.1, CP027852.1, CP076372.1, CP050969.1, OR138057.1, CP076371.1, CP062196.1, DQ814804.1, HM778593.1, DQ814533.1
13	*Proteus vulgaris*	99.65%	KP313869.1	0.0	100.00%	KP313869.1, CP095785.1, GU812899.1, MG027634.1, CP065722.1, KP313867.1, AB682277.1, EF426446.1, MG438540.1, KP313868.1
14	*Pseudomonas aeruginosa*	100.00%	FJ972533.1	0.0	100.00%	FJ972533.1, OQ678887.1, KT285549.1, MT373467.1, KX860116.1, HM587311.1, KF113578.1, MT197312.1, OR673041.1, MF401349.1
15	*Pseudomonas parafulva*	100.00%	CP160620.1	0.0	100.00%	CP160620.1, CP130566.1, CP023048.1, CP161879.1, CP132347.1, MW672531.1, AB480754.1, NR040859.1, AM411621.1, CP191382.1
16	*Pseudomonas putida*	100.00%	OQ411307.1	0.0	100.00%	OQ411307.1, CP156901.1, KX350025.1, CP062699.1, CP005976.1, KF841255.1, PP262582.1, OP627904.1,

						CP132350.1, MK824529.1
17	*Shewanella xiamenensis*	99.93%	AP025014.1	0.0	100.00%	AP025014.1, MG428836.1, CP091833.1, KY928091.1, HM779405.1, MG428831.1, JX828428.1, MG428824.1, HM778832.1, CP022089.2
18	*Streptococcus agalactiae*	99.07%	KU561093.1	0.0	100.00%	KU561093.1, JQ039367.1, JQ039375.1, JQ039360.1, JQ771298.1, OX460968.1, CP157196.1, PP939757.1, PP939767.1, PP939772.1
19	*Vibrio alginolyticus*	99.79%	JX913856.1	0.0	100.00%	JX913856.1, KJ841878.1, MZ027052.1, MG996624.1, AF500207.1, CP054700.1, CP017919.1, CP169050.1, CP148900.1, CP014053.1
20	*Vibrio cholerae*	99.93%	CP053802.1	0.0	100.00%	CP053802.1, CP053822.1, CP022353.1, CP104356.1, CP137093.1, CP046740.1, EF684901.1, MF692792.1, CP028892.1, CP053816.1
21	*Vibrio parahaemolyticus*	100.00%	HG999491.1	0.0	100.00%	HG999491.1, KR270231.1, KR270273.1, KR270318.1, KR270194.1, KR270232.1, KR270241.1, KR270217.1, KR270247.1, KR270301.1

2

Supplementary Table S2. The BLAST results for gyrB gene of the isolated strains.

S. No.	Bacterial species	Percentage identity	Accession numberof the closest match	E-value	Query coverage	Reference sequences used for phylogenetic tree construction
1	*Aeromonas caviae*	100.00%	MW659695.1	0.0	100.00%	MW659695.1, KJ747119.1, OP121106.1, CP025777.1, MN855479.1, CP025706.1, AY987527.1, MG751872.1, AB473060.1, JQ234886.1
2	*Aeromonas dhakensis*	98.22%	AP028321.1	0.0	100.00%	AP028321.1, CP102365.1, JN602730.1, AY987502.1, AY987507.1, AY987501.1, JN602729.1, MT967985.1, CP070217.1, CP084349.1
3	*Aeromonas hydrophila*	100.00%	DQ519366.1	0.0	100.00%	DQ519366.1, CP119431.1, CP102327.1, CP054854.1, AP027942.1, AP028321.1, OY731358.1, AP027936.1, CP102326.1, OY731370.1
4	*Aeromonas jandaei*	100.00%	OP121111.1	0.0	100.00%	OP121111.1, OQ743463.1, OQ743464.1, CP084376.1, AJ868391.1, CP149571.1, CP066092.1, CP070220.1, AY987511.1, CP043322.1
5	*Aeromonas salmonicida*	97.41%	CP102172.1	0.0	100.00%	CP102172.1, CP149127.1, CP110645.1, CP101948.1, CP091480.1, OM203113.1, CP080042.1, CP060030.1,

						CP047374.1, CP116258.1
6	*Aeromonas veronii*	100.00%	MK415380.1	0.0	100.00%	MK415380.1, CP077221.1, CP121816.1, CP024930.1, MW838041.1, MW838056.1, CP080630.1, CP121817.1, MW838078.1, CP028133.1
7	*Chryseobacterium indologenes*	95.68%	CP073027.1	0.0	100.00%	CP073027.1, CP045734.1, CP134622.1, CP068760.1, CP018786.1, CP120710.1, CP132966.1, CP143637.1, CP035532.1, CP045735.1
8	*Edwardsiella piscicida*	100.00%	JX867004.2	0.0	100.00%	JX867004.2, CP094304.2, CP094311.2, CP095163.1, CP094315.2, CP094324.2, CP006664.1, CP011516.2, CP094313.2, CP094302.2
9	*Enterococcus faecalis*	100.00%	GQ426104.1	0.0	100.00%	GQ426104.1, CP185997.1, CP046112.1, LR962695.1, CP157638.1, CP157641.1, CP124947.1, LR962190.1, LR961945.1, LR962101.1
10	*Lactococcus garvieae*	94.36%	GU324261.1	0.0	100.00%	GU324261.1, CP109635.1, CP141703.1, CP146749.1, CP086401.1, CP146754.1, CP120417.1, CP146738.1, CP174196.1, CP109636.1
11	*Photobacterium damselae*	100.00%	KU680722.1	0.0	100.00%	KU680722.1, CP073684.1, CP070624.1, KU680718.1, CP079237.1, CP146973.1, KU680715.1, CP099545.1,

						AY455889.1, AJ249850.1
12	*Plesiomonas shigelloides*	100.00%	OP222557.1	0.0	100.00%	OP222557.1, CP050969.1, CP062196.1, GQ426109.1, CP101030.1, CP087711.1, OP222555.1, OP222554.1, CP076371.1, DQ316983.1
13	*Proteus vulgaris*	100.00%	AJ300544.1	0.0	100.00%	AJ300544.1, LR590468.1, CP083628.1, CP033736.1, CP104121.1, CP166027.1, MK119782.1, CP047639.1, CP065722.1, CP059690.1
14	*Pseudomonas aeruginosa*	100.00%	FJ652721.1	0.0	100.00%	FJ652721.1, CP031659.1, CP039990.1, CP050148.1, CP123953.1, CP051547.1, CP050323.1, CP075841.1, CP047697.1, CP029713.1
15	*Pseudomonas parafulva*	100.00%	FJ418638.1	0.0	100.00%	FJ418638.1, FN554216.1, CP050951.1, CP087183.1, CP151298 1, CP092827.1, CP151299.1, CP140007.1, CP157873.1, CP144123.1
16	*Pseudomonas putida*	100.00%	MK690197.1	0.0	100.00%	MK690197.1, AP023348.2, CP087165.1, CP034337.1, GU354315.1, MH431555.1, CP034338.1, CP087185.1, CP081016.1, PV391151.1
17	*Shewanella xiamenensis*	98.32%	FJ589040.1	0.0	100.00%	FJ589040.1, CP106874.1, CP022089.2, EU492928.1, KC765141.1, CP069350.1, AP025014.1, FJ589044.1,

						CP079718.1, CP143633.1
18	*Streptococcus agalactiae*	100.00%	LC649415.1	0.0	100.00%	LC649415.1, CP033808.1, CP169989.1, CP007631.2, CP021773.1, CP021772.1, CP031556.1, CP053889.1, CP042002.1, LC649412.1
19	*Vibrio alginolyticus*	100.00%	PP072268.1	0.0	100.00%	PP072268.1, CP013484.1, CP060386.1, CP016224.1, CP042449.1, LR860665.1, CP076287.1, EU680781.1, CP014045.1, EF579670.1
20	*Vibrio cholerae*	97.12%	MT512616.1	0.0	100.00%	MT512616.1, MN784639.1, CP046737.1, AP023373.1, CP189226.1, CP188870.1, CP189254.1, CP188993.1, CP152071.1, CP093426.1
21	*Vibrio parahaemolyticus*	99.31%	FJ847647.1	0.0	100.00%	FJ847647.1, CP035701.1, CP046763.1, EU051574.1, FJ847598.1, CP006004.1, CP078637.1, HM009557.1, FJ577416.1, CP023710.1
